# Patient safety culture in home care settings in Sweden: a cross-sectional survey among home care professionals

**DOI:** 10.1186/s12913-023-10010-y

**Published:** 2023-09-16

**Authors:** Anastasia Silverglow, Helle Wijk, Eva Lidén, Lena Johansson

**Affiliations:** 1 Institute of Health and Care Sciences at Sahlgrenska Academy, University of Gothenburg, Gothenburg, Sweden; 2 Sahlgrenska University Hospital, Gothenburg, Sweden; 3 The Centre for Healthcare Architecture (CVA), Chalmers University of Technology, Gothenburg, Sweden; 4 Department of Psychiatry and Neurochemistry, Sahlgrenska Academy, Centre for Ageing and Health (AgeCap) at the University of Gothenburg, Gothenburg, Sweden

**Keywords:** Patient safety culture, Home care, HSOPSC, Cross-sectional study

## Abstract

**Background:**

The connection between a weak patient safety culture and adverse patient events is well known, but although most long-term care is provided outside of hospitals, the focus of patient safety culture is most commonly on inpatient care. In Sweden, more than a third of people who receive care at home have been affected by adverse events, with the majority judged to be preventable. The aim of this study was to investigate the patient safety culture among care professionals working in care at home with older people.

**Methods:**

This cross-sectional study used a purposive sample of 66 municipal care workers, health care professionals, and rehabilitation staff from five municipal care units in two districts in western Sweden who provided care at home for older people and had been employed for at least six months. The participants completed the Hospital Survey on Patient Safety Culture (HSOPSC) self-report questionnaire, which assessed aspects of patient safety culture—norms, beliefs, and attitudes. Logistic regression analysis was used to test how the global ratings of *Patient safety grade* in the care units and *Reporting of patient safety events* were related to the dimensions of safety culture according to the staff’s professions and years of work experience.

**Results:**

The most positively rated safety culture dimension was *Teamwork within care units* (82%), which indicates good cooperation with the closest co-workers. The least positively rated dimensions were *Handoffs and transitions among care units* (37%) and *Management support* (37%), which indicate weaknesses in the exchange of patient information across care units and limited support from top-level managers. The global rating of *Patient safety grade* was associated with *Communication openness* and *Management support* (*p* < 0.01 and *p* = 0.03, respectively). Staff with less work experience evaluated the *Patient safety grade* higher than those with more work experience.

**Conclusions:**

This study suggests that improvements are needed in care transitions and in support from top-level managers and that awareness of patient safety should be improved in staff with less work experience. The results also highlight that an open communication climate within the care unit is important for patient safety.

**Supplementary Information:**

The online version contains supplementary material available at 10.1186/s12913-023-10010-y.

## Background

Patient safety, in terms of the prevention of adverse events in health care, is a key component of quality of care [1–3]. During the last few decades, patient safety research has extended from focusing solely on human errors to include organization-related issues [4–6]. The awareness of a relationship between a weak patient safety culture and adverse events [4, 7–9] has thus been an impetus for research on safety culture, which has focused on norms, beliefs, attitudes, and perceptions among professionals, which are assumed to affect their collective values and patterns of behavior [10, 11].

Although most long-term care is provided outside the hospital [12], the focus of patient safety culture—the aspects of safety culture that relate specifically to patient safety—is most commonly on inpatient care [13, 14]. Health care policies in Sweden support aging in place through the provision of care at home [15] based on multi-profession teamwork [16, 17], embracing a cooperation between licensed nurses, physiotherapists, occupational therapists [18, 19], and unlicensed staff who provide homemaker services. These home care staff have different roles, duties, and responsibilities in care recipients’ homes [20], and they may be employed by any of three different municipal care organizations, each governed by different legislation and using different documentation systems [21, 22], which all affect opportunities for cooperation. Employees also usually work alone in the private space of a person’s home [23, 24] and must therefore seek to enable patient autonomy in a safe way [23, 25]. Furthermore, receivers of care at home are mainly older people, who are particularly vulnerable for reasons that include multi-morbidity [26], polypharmacy [27], and unmet needs [28].

In Sweden, more than 30% of people who receive care at home have been affected by adverse events, with a majority judged to be preventable [29]. In this setting, registered nurses have a crucial role in preventing no-harm incidents and adverse events [30], and the large number of adverse events demands research into their causes as well as into the enablers of safe care in the home context. An investigation of patient safety culture in care at home could provide a basis for improvements [4, 8], but as far as we know, there have been no studies on this topic in Sweden. Some studies into care at home in other countries have considered teamwork [31, 32], supervisor expectations, and communication about incidents [32] as valuable dimensions of a well-functioning patient safety culture, while weaknesses have been reported in staff resources [32] and support from management [33]. These previous studies have also illuminated the complexity and difficulty of recruiting a sufficiently large number of informants to conduct quantitative studies in home care settings [31–33], which indicates the need for more studies in this field to provide a sufficient basis for patient safety improvements.

Higher staff educational levels [34, 35] and less work experience among nurses (≤ 5 years) [36] are strongly connected with fewer adverse events in hospital settings. However, similar studies in the home care context are lacking, which presents particular issues because unlicensed staff—those without higher education in health care disciplines—are the largest group of professionals providing care at home [37].

A starting point for improving patient safety in a health care organization is to increase consciousness of patient safety culture [3, 38, 39]. This study therefore aimed to investigate the patient safety culture among care professionals working in care at home with older people. More specifically, the objectives were 1) to describe the most and least positively ranked patient safety culture dimensions; 2) to compare ratings between unlicensed and licensed care professionals and between those with more and less work experience; and 3) to analyze associations between patient safety culture dimensions and both the global rating of patient safety grade and the reporting of patient safety events.

## Methods

### Data collection

This cross-sectional study used a purposive sample of care professionals providing care at home for older people in five municipal care units in two districts—one urban and one rural—in western Sweden and was conducted between December 2018 and February 2019. Initially, to get in touch with potential participants, the first author provided study information to municipal care unit managers and asked them for permission to conduct the study in their care units. Then, at the municipal care units for which permission had been obtained from the care unit managers, oral and written study information, including the voluntary nature of any participation, was provided by the first author to the employees. Finally, the Hospital Survey on Patient Safety Culture (HSOPSC) questionnaire [40, 41] was distributed by the first author to all municipal care workers, health care professionals, and rehabilitation staff who had been employed for at least six months in the two districts and who gave consent to participate in the study. The questionnaires were completed individually and on paper at the participants’ workplaces; participation was voluntary, all answers were confidential, and no care unit managers were present while the care professionals completed the forms. A total of 80 questionnaires were administered, of which 66 were completed (response rate 83%). The care professionals who did not answer the questionnaires stated that they had a shortage of time (*n* = 8), had changed their minds about participating (*n* = 2), or had terminated their employment (*n* = 4). A care unit was defined for the participants as being a part of a municipal care organization, having one care unit manager, being staffed by a regular group of care professionals, being restricted to a delimited geographical district, and providing care at home for care recipients living in that district.

#### HSOPSC and dimensions of patient safety culture

HSOPSC is a well-established instrument that was developed by the U.S. Agency for Healthcare Research and Quality [41] to investigate norms, beliefs, attitudes, and perceptions among professionals [11]. It has been recognized and used in more than 45 countries [42] and translated into more than 20 languages. In Sweden, the HSOPSC has been validated for use in both small [43] and large [40] samples. In the current study, the Swedish adaptation of the questionnaire, prepared by Hedsköld et al. [40], was used, with minor changes to the wording to adapt it to the home care context, such as “older person” instead of “patient” and “care units” instead of “hospital units.”

Eleven patient safety dimensions were constructed from the 39 questions of the HSOPSC instrument [41] (see Additional file 1). Seven dimensions were related to internal staff perceptions of the working group: 1) *Staffing resources*; 2) *Communication openness*; 3) *Teamwork within care units*; 4) *Supervisor/manager expectations and actions promoting safety*; 5) *Non-punitive response to errors*; 6) *Feedback and communication about errors*; and 7) *Overall perceptions of safety*. Four dimensions were related to perspectives external to the workgroup: 1) *Teamwork across care units*; 2) *Handoffs and transitions among care units*; 3) *Organizational learning—continuous improvement*; and 4) *Management support for patient safety*.

Each dimension was based on three or four questions, which were scored by participants on a five-point Likert scale of agreement (1 = “strongly disagree” to 5 = “strongly agree”) [41]. The responses “agree/strongly agree” indicated a positive assessment of patient safety for positively worded items, while “disagree/strongly disagree” indicated a positive assessment of patient safety for negatively worded items. A higher percentage of positive assessments indicates a better patient safety culture, and this percentage is presented as the average positive response (APR) [41], which is a measure that has been used and described in previously published studies [44, 45].

#### Global rating of patient safety grade and reporting of patient safety events

The global rating of *Patient safety grade* was measured with a single item (“Please give your care unit an overall grade on patient safety”) and was rated on a five-point Likert scale from 1 = “failing” to 5 = “excellent” [41]. *Reporting of patient safety events* assesses care professionals’ reports of mistakes, risks, or adverse events and consists of three items: 1) When a mistake is made but is caught and corrected before affecting the patient, how often is this reported? 2) When a mistake is made but has no potential to harm the patient, how often is this reported? 3) When a mistake is made that could harm the patient, but it does not, how often is this reported? It was measured on a five-point Likert scale of frequency, from 1 = “never” to 5 = “always” [41].

#### Demographics

The study also assessed several demographic characteristics: sex, age, level of profession (licensed versus unlicensed staff), years of work experience in health care (total length of work experience in his or her care profession), years working at the current unit (≤ 5 years or > 5 years), and occupational group (municipal care workers, health care professionals, rehabilitation staff).

### Statistical analyses

Analyses were conducted using SPSS Statistics 25 (IBM, Armonk, New York, USA) [46]. All data were anonymized using code numbers for the individual questionnaires. Descriptive analyses were used to present the respondents’ background characteristics and explore patient safety culture dimensions among the staff. The APR per dimension was calculated by adding the percentage of positive responses on all items and dividing it by the number of items in the corresponding dimension [41]. Independent samples t-tests were conducted to evaluate whether the ratings of the patient safety culture dimensions varied according to the levels of profession (licensed or unlicensed staff) or years of experience working in health care (≤ 5 years or > 5 years).

Linear regression analyses were performed to explore the relationship between the 11 patient safety culture dimensions (as predictors) and both the global rating of *Patient safety grade* and *Reporting of patient safety events* (as outcomes). *Patient safety grade* was first analyzed against each dimension individually and then by including all 11 dimensions in the same model, using the SPSS “Enter” option. The analyses were repeated with *Reporting of patient safety events* as the outcome variable. All regression analyses were adjusted to the respondents’ professions and years of experience working in health care.

The results are presented with the R^2^, F(df), β, 95% CI, and *p*-values. The internal consistencies of the dimensions of the HSOPSC were assessed using Pearson correlation coefficients and are reported with 95% CI and *p*-values (Additional file 2). In all cases, a *p*-value of less than 0.05 was considered to be statistically significant.

## Results

A majority of the sample (*n* = 66) were female (82%) and a majority were older than 35 years (75%). Twenty-four were licensed staff and 42 were unlicensed, with 45 having more than five years of work experience in health care (79% of licensed staff, 60% of unlicensed staff) (Table 1).

**Table 1:** Demographic characteristics of respondents

Characteristics	Licensed staff n (%)	Unlicensed staff n (%)	Total sample n (%)
Sex			
Female	22 (91.7)	32 (76.2)	54 (81.8)
Male	2 (8.3)	10 (23.8)	12 (18.2)
Age			
18–34 years	4 (16.7)	12 (30.0)	16 (25.0)
35–54 years	13 (54.2)	21 (52.5)	34 (53.1)
≥ 55 years	7 (29.2)	7 (17.5)	14 (21.9)
Missing	0	2	2
Profession			
Care assistant	0	17 (40.5)	17 (25.8)
Nursing assistant	0	19 (45.2)	19 (28.8)
Registered/specialist nurse	16 (66.7)	0	16 (24.2)
Physiotherapist	4 (16.7)	0	4 (6.1)
Occupational therapist	4 (16.7)	0	4 (6.1)
First-line manager	0	3 (7.1)	3 (4.5)
Other^a^	0	3 (7.1)	3 (4.5)
Work experience in health care			
≤ 5 years	5 (20.8)	16 (40.0)	21 (32.8)
6–10 years	2 (8.3)	6 (15.0)	8 (12.5)
≥ 11 years	17 (70.8)	18 (45.0)	35 (54.7)
Missing	0	2	2
Work experience at current workplace			
≤ 5 years	13 (54.2)	30 (75.0)	43 (67.2)
6–10 years	6 (25.0)	6 (15.0)	12 (18.8)
≥ 11 years	5 (20.8)	4 (10.0)	9 (14.1)
Missing	0	2	2
Work team			
Municipal care workers	0	41 (97.6)	41 (62.1)
Health care professionals	18 (75.0)	1 (2.4)	19 (28.8)
Rehabilitation staff	6 (25.0)	0	6 (9.1)

^a^Care administrator, method instructor, and coordinator

The ratings of the 11 safety culture dimensions varied, and the APRs ranged from 37 to 82%. The most positively rated dimension was *Teamwork within care units* (82%), with the participants reporting a high degree of support and cooperation with their closest co-workers (Table 2).

**Table 2:** Respondent perceptions of patient safety culture dimensions

Safety culture dimensions	Total		Licensed staff		Unlicensed staff		*p*-value^a^
	APR*(%)	**Mean ± SD**	**APR (%)**	**Mean ± SD**	**APR (%)**	**Mean ± SD**	
Staffing resources	49.3	3.27 ± 0.91	72.9	3.98 ± 0.74	35.0	2.86 ± 0.74	< 0.001
Communication openness	48.3	3.56 ± 0.74	38.7	3.23 ± 0.71	54.0	3.75 ± 0.70	0.007
Teamwork within care units	82.4	3.97 ± 0.57	81.0	4.06 ± 0.66	83.2	3.92 ± 0.51	0.372
Supervisor/manager expectations and actions promoting safety	58.7	3.66 ± 0.65	52.7	3.52 ± 0.71	62.4	3.74 ± 0.61	0.224
Non-punitive response to error	41.6	3.30 ± 0.10	55.5	3.70 ± 0.85	34.0	3.08 ± 0.99	0.012
Feedback and communication about error	54.4	3.62 ± 0.83	23.8	2.99 ± 0.65	71.1	3.96 ± 0.71	< 0.001
Overall perceptions of safety	52.7	3.44 ± 0.77	59.6	3.63 ± 0.89	48.2	3.34 ± 0.68	0.184
Teamwork across care units	48.2	3.38 ± 0.55	40.9	3.20 ± 0.43	52.1	3.47 ± 0.58	0.046
Handoffs and transitions among care units	36.5	3.07 ± 0.75	48.7	3.37 ± 0.57	28.7	2.90 ± 0.79	0.010
Organizational learning—continuous improvement	53.0	3.53 ± 0.77	34.4	3.10 ± 0.85	62.4	3.78 ± 0.61	0.002
Management support for patient safety	36.5	3.21 ± 0.73	28.8	3.03 ± 0.71	41.1	3.31 ± 0.72	0.153

^*^Average positive response

^a^Independent samples t-test

The least positively rated dimensions were *Handoffs and transitions among care units* (37%) and *Management support* (37%), i.e., the participants estimated patient safety as low in terms of the exchange of patient information across care units and support from their top-level managers in providing a work climate that promotes patient safety. For seven of the dimensions, the ratings differed between unlicensed and licensed staff (Table 2).

The mean overall evaluation of *Patient safety grade* at the care unit was 3.52 (SD = 0.74) on a 5-point scale. The outcome *Patient safety grade* was significantly predicted by two dimensions: *Communication openness* (β = 0.40, *p* < 0.01) and *Management support for patient safety* (β = 0.30, *p* = 0.03) (Table 3). Staff with less work experience (≤ 5 years) rated the *Patient safety grade* higher than those with more work experience (> 5 years) (3.84 ± 0.76 and 3.38 ± 0.70, respectively; *p* = 0.03), but there were no differences between licensed and unlicensed staff.

**Table 3:** Multiple linear regression analyses of relationships between patient safety culture dimensions and the global *Patient safety grade* rating

**Safety culture dimensions**^**a**^	**Global Patient safety grade rating**^**b**^					**Global Patient safety grade rating**^**c**^ ***n*** = 53**; R**^**2**^ = 0.51; **F (df)** = 3.11 (13)	
	**n**	**R**^**2**^	**F (df)**	**β (95% CI)**	***p*-value**	**β (95% CI)**	***p*-value**
Staffing resources	61	0.11	2.25 (3)	0.17 (-0.15 to 0.50)	0.30	-0.10 (-0.51 to 0.31)	0.64
Communication openness	60	0.29	7.43 (3)	0.53 (0.26 to 0.79)	< 0.01	0.54 (0.18 to 0.89)	< 0.01
Teamwork within care units	61	0.17	3.88 (3)	0.29 (0.04 to 0.53)	0.02	0.09 (-0.21 to 0.38)	0.55
Teamwork across care units	57	0.20	4.44 (3)	0.36 (0.10 to 0.63)	0.01	0.13 (-0.22 to 0.48)	0.46
Handoffs and transitions among care units	56	0.10	1.67 (3)	0.02 (-0.28 to 0.33)	0.88	-0.15 (-0.47 to 0.17)	0.35
Supervisor/manager expectations and actions promoting safety	60	0.10	2.07 (3)	0.13 (-0.13 to 0.40)	0.33	0.00 (-0.40 to 0.39)	0.10
Non-punitive response to error	58	0.09	1.84 (3)	0.64 (-0.23 to 0.36)	0.67	-0.20 (-0.56 to 0.16)	0.27
Feedback and communication about error	60	1.18	4.11 (3)	0.39 (0.09 to 0.70)	0.01	0.08 (-0.37 to 0.52)	0.73
Organizational learning—continuous improvement	59	0.19	4.40 (3)	0.38 (0.10 to 0.65)	0.01	-0.06 (-0.51 to 0.38)	0.77
Management support for patient safety	55	0.31	7.71 (3)	0.52 (0.26 to 0.78)	< 0.01	0.40 (0.04 to 0.75)	0.03
Overall perceptions of safety	60	0.21	5.02 (3)	0.39 (0.13 to 0.65)	< 0.01	0.07 (-0.34 to 0.47)	0.74

^a^Each dimension was based on a five-point Likert scale

^b^The analyses include each dimension individually with *Patient safety grade*

^c^The analyses include all 11 dimensions and *Patient safety grade* in the same model

^bc^All analyses were adjusted for the level of profession (licensed or unlicensed staff) and years of experience working in health care (≤ 5 years or > 5 years)

The mean rating of *Reporting patient safety events* was 2.67 (SD = 0.98). This outcome was significantly predicted by two dimensions: *Feedback and communication about errors* (β = 0.49, *p* < 0.01) and *Management support for patient safety* (β = 0.49, *p* < 0.01) (Table 4). Participants with less work experience (*p* = 0.02) and unlicensed staff (*p* = 0.04) gave higher *Reporting patient safety events* ratings than those with more work experience and those who were licensed, respectively.

**Table 4:** Multiple linear regression analyses of relationships between patient safety culture dimensions and the global *Reporting patient safety events* rating

**Safety culture dimensions**^a^	**Global Reporting patient safety events rating**^b^					**Global Reporting patient safety events rating**^c^ ***n* = **51;** R**^**2**^ = 0.64; **F (df)** = 4.10 (13)	
	**n**	**R**^**2**^	**F (df)**	**β (95% CI)**	***p*-value**	**β (95% CI)**	***p*-value**
Staffing resources	56	0.14	2.81 (3)	-0.07 (-0.42 to 0.27)	0.68	-0.10 (-0.45 to 0.25)	0.56
Communication openness	56	0.16	3.38 (3)	0.19 (-0.11 to 0.49)	0.21	0.08 (-0.21 to 0.38)	0.57
Teamwork within care units	56	0.17	3.57 (3)	0.19 (-0.07 to 0.45)	0.15	0.06 (-0.19 to 0.31)	0.62
Teamwork across care units	54	0.20	4.24 (3)	0.21 (-0.04 to 0.46)	0.10	-0.11 (-0.40 to 0.17)	0.43
Handoffs and transitions among care units	53	0.16	3.16 (3)	0.07 (-0.21 to 0.36)	0.62	0.06 (-0.21 to 0.32)	0.66
Supervisor/manager expectations and actions promoting safety	56	1.14	2.76 (3)	0.03 (-0.23 to 0.29)	0.82	-0.27 (-0.59 to 0.06)	0.11
Non-punitive response to error	56	1.14	2.76(3)	0.03 (-0.26 to 0.31)	0.84	-0.03 (-0.33 to 0.26)	0.82
Feedback and communication about error	56	0.43	12.90 (3)	0.65 (0.40 to 0.91)	< 0.01	0.58 (0.20 to 0.97)	< 0.01
Organizational learning—continuous improvement	55	0.27	6.12 (3)	0.46 (0.15 to 0.76)	0.01	-0.12 (-0.53 to 0.29)	0.56
Management support for patient safety	52	0.39	10.39 (3)	0.51 (0.27 to 0.74)	< 0.01	0.49 (0.21 to 0.78)	< 0.01
Overall perceptions of safety	56	0.14	2.75 (3)	-0.02 (-0.33 to 0.30)	0.91	-0.01 (-0.37 to 0.34)	0.94

^a^Each dimension was based on a five-point Likert scale

^b^The analyses include each dimension individually with *Patient safety grade*

^c^The analyses include all 11 dimensions and *Patient safety grade* in the same model

^bc^All analyses were adjusted for the level of profession (licensed or unlicensed staff) and years of experience working in health care (≤ 5 years or > 5 years)

## Discussion

The findings indicate that the participants perceived a well-functioning patient safety culture in relation to *Teamwork within care units*, but there were limitations to cooperation between units and limited support from top-level managers. *Management support* was very valuable, both for the reported *Patient safety grade* within care units and for the reporting of mistakes, risks, or adverse events. In addition, *Communication openness* was associated with higher reported *Patient safety grade*, while *Feedback and communication about errors* was associated with better *Reporting patient safety events*. Staff with shorter work experience and unlicensed staff reported a higher *Patient safety grade* and better *Reporting patient safety events*.

Teamwork is described as one of the six core competencies for health care professionals that are needed to provide safe and high-quality care [47]. Effective teamwork requires understanding of and respect for the roles and competencies of others within a care organization as well as ongoing communication, trust, willingness to collaborate, and feeling other team members’ support [48]. Thus, the finding that mutual help, support, and respect among home care staff is scored highly within care units should be used as a starting point to improve other dimensions of patient safety culture. To accomplish this in the home care setting, the Agency for Healthcare Research and Quality's structured approach to teamwork—Team Strategies and Tools to Enhance Performance and Patient Safety (TeamSTEPPS)—which focuses on team awareness and team members’ roles and responsibilities, with a focus on improving safety and quality of care, could be suitable [49].

The participants reported dissatisfaction with the exchange of information between care units. This finding highlights staff perceptions of insufficient cooperation across professional and organizational boundaries, which is a well-known issue in home care settings [27, 50] and a risk to patient safety. Cooperation in care at home is challenging because of the involvement of multiple caregivers [15, 18–20] and the differences in structures and processes, such as scheduling of care, documentation systems, and professional roles in home care settings [23, 51], all of which further increase the risks of gaps in care responsibilities and the restriction or absence of technical aids [23]. Poor cooperation between organizations can also lead to a broad range of incidents, with falls and medication management failures having been identified as the most common and potentially preventable issues [30, 50]. Insufficient communication between different care providers in home care settings has been shown to be a severe risk factor for medication-related problems, such as incorrect dosages or durations, inconsistencies in a patient’s medication lists, and adverse reactions due to drug interactions [27, 52–54]. This indicates a need for massive efforts to improve cooperation between care organizations and care professionals. In this setting, joint meetings or team meetings could improve mutual understanding of each other’s competences, increase mutual trust, and improve preventive work. A common documentation system could also help prevent gaps in care responsibilities [23].

The study found that *Management support* was both one of the least positively scored dimensions of patient safety culture and important for the overall perceived *Patient safety grade* and for *Reporting patient safety events*, which is partly in line with previous research conducted in nursing homes [55] and hospital care settings [45, 56]. Top-level managers have an important role in maintaining and improving patient safety in care units and in supporting staff in working safely [57]. Reporting systems require staff awareness of the reporting approach and an environment that facilitates the disclosure of failures and protects the staff involved [58]. These findings indicate a need to find effective strategies to improve leadership in these areas and to increase managers’ awareness of the importance of risk reporting [59].

We also found that *Communication openness* was the strongest predictor of higher *Patient safety grade* while *Feedback and communication about errors* was —in line with a study by Ammouri et al. [56]—one of the strongest predictors of increased *Reporting of patient safety events,* which shows the importance of mutual communication between staff and first-line managers in a care unit. Mutual communication is perceived as one of the crucial prerequisites for providing safe care outside the hospital because it allows each professional to understand the others’ competences and involvement and to formulate care continuity [23, 60]. The findings of the present study demonstrate that communication problems, both between care professionals and with care unit managers, can weaken patient safety in home care. Thus, this study calls for proactive leadership at all care management levels that is characterized by functional routines for reporting patient safety events, effective feedback, and mutual trust, which would increase care professionals’ influence on patient safety work in care at home.

The current study found that staff with more work experience gave a lower *Patient safety grade* and had a lower grade for risks, mistakes, and adverse event reporting, which is in line with a study by Tomazoni et al. [61]. Having more competence could also be connected to having a license, with licensed staff giving a lower event reporting grade than unlicensed staff. These findings could be related to the staff’s ability to think critically, which is an essential skill for providing safe care and maintaining competence [62]. Critical thinking improves the ability of care professionals to work proactively by being prepared for issues associated with patient care in advance. This approach reduces feelings of unsafety, which in turn reduces patient safety risks [63] and may lead to less error reporting. At the same time, as in this study, critical thinking may increase staff safety awareness, which could lead to a lower score for *Patient safety grade* from staff with longer work experience. The availability of educational opportunities and an environment that enables care professionals’ participation in decision-making processes are extremely important factors for developing critical thinking [64]. In practice, this emphasizes the need for functional resources and decision support through multi-professional teams [65] as well as new strategies in education that would offer students a different perspective and understanding of care processes [64].

The findings showed that licensed and unlicensed staff scored differently in a majority (seven of 11) of the safety culture dimensions. The lowest assessments were found among unlicensed staff in relation to information transitions between different care units, the opportunity to talk about errors without fear of punishment, and staff resources. Unlicensed staff need to perform complex tasks and manage unexpected situations, including older people’s personal care or the administration of medication, while they are working alone [23]. Their decision-making regarding how to perform care at home for each individual requires insight into each person’s needs and practical circumstances [66]. The findings showing the vulnerability of unlicensed staff call attention to the need for interprofessional collaborations, organizational clarity [65], and the support and supervision of multidisciplinary team members [23].

### Strengths and limitations

The strength of this study is that it illuminates patient safety culture in the home care setting, which has been unexplored in the Swedish population. Although the original questionnaire has good psychometric properties [41], it was not designed for use in care at home. Nevertheless, the HSOPSC is one of the most used patient safety culture instruments in community care [63]; beyond hospital care settings, it has been previously used in primary care [38] and in elderly care homes [67, 68] and was considered user-friendly in this study in the sense that the participants recognized the relevance of the questions and did not report difficulties in completing the questionnaire. This study therefore provides important knowledge relating to the usefulness of the questionnaire in home care settings.

The HSOPSC is also the only questionnaire investigating patient safety culture that has been translated into Swedish and validated in Swedish health care settings [40]. Although the study provides insight into the perceptions of patient safety culture in three groups of home care professionals, the findings cannot be generalized due to the small sample, although small samples have also been used in previous studies in this field [31–33], illustrating the difficulties of conducting quantitative studies in home care settings. The authors are aware of the low statistical power in this study and sought to recruit more participants by contacting other municipal care units in the participating districts and in other districts. Unfortunately, all the other municipal care unit managers who were contacted reported large staff turnover and a lack of staff time to participate. However, the variability in the respondents’ demographic characteristics in terms of profession, occupational group, and workplace (urban/rural area) is representative of home care staff across Sweden, and the overall response rate of 83% ensured an adequate representation of staff views [41]. In addition, the study allows comparability with future studies that use the HSOPSC in similar care settings.

The current study was completed before the COVID-19 pandemic, and some dimensions of patient safety culture may appear differently after the pandemic. Even so, this research remains highly relevant because of the need to investigate the possible positive or negative impacts of the pandemic on patient safety culture in home care. In this context, the current study also provides a valuable basis for post-pandemic comparisons.

Future research should be conducted to test the Swedish adaptation of HSOPSC on a larger sample in home care settings. The relationships between patient safety culture in home care settings and patient outcomes should also be investigated.

## Conclusion

The study suggests improvements are needed in care transitions, support from top-level managers, and awareness of patient safety among staff with less work experience. The results also highlighted that an open and accommodating communication climate in a care unit is important for patient safety. To maintain and improve teamwork, an evidence-based framework like TeamSTEPPS could be applied in this care setting; team meetings and a common documentation system are also critical for providing safe care at home. A functional system for reporting patient safety events requires proactive leadership at all care management levels and would enable care professionals to influence patient safety work. Finally, the educational system should embrace innovative teaching methods to improve the ability of future care professionals to think proactively and critically.

### Supplementary Information

**Additional file 1.** Safety culture dimensions and items^a^.

**Additional file 2.** Correlation matrix.

## Data Availability

The datasets generated and/or analyzed during the current study are not publicly available due to their containing information that could compromise the privacy of research participants but are available from the corresponding author on reasonable request.
