# Supplementary material for: Patient safety culture in home care settings in Sweden: a cross-sectional survey among home care professionals
Source: BMC Health Serv Res. 2023 Sep 16;23:998. doi: 10.1186/s12913-023-10010-y (PMC10505324; doi:10.1186/s12913-023-10010-y)
Supplement: Supplementary file 1 — Additional file 1. Safety culture dimensions and itemsa. [file 12913_2023_10010_MOESM1_ESM.docx]

**Additional file 1**

**Safety culture dimensions and items ^a^**

| **Safety culture dimensions** |
| --- |
| **Staffing** |
| We have enough staff to handle the workload |
| Staff in this unit work longer hours than is best for patient care **^b^** |
| We use more agency/temporary staff than is best for patient care **^b^** |
| We work in “crisis mode,” trying to do too much, too quickly **^b^** |
| **Communication openness** |
| Staff will freely speak up if they see something that may negatively affect patient care |
| Staff feel free to question the decisions or actions of those with more authority |
| Staff are afraid to ask questions when something does not seem right **^b^** |
| **Teamwork within care units** |
| People support one another in this unit |
| When a lot of work needs to be done quickly, we work together as a team to get the work done |
| In this unit, people treat each other with respect |
| When one area in this unit gets really busy, others help out |
| **Supervisor/manager expectations and actions promoting safety** |
| My supervisor/manager says a good word when he/she sees a job done according to established patient safety procedures |
| My supervisor/manager seriously considers staff suggestions for improving patient safety |
| Whenever pressure builds up, my supervisor/manager wants us to work faster, even if it means taking shortcuts **^b^** |
| My supervisor/manager overlooks patient safety problems that happen over and over **^b^** |
| **Non-punitive response to errors** |
| Staff feel like their mistakes are held against them **^b^** |
| When an event is reported, it feels like the person is being written up, not the problem **^b^** |
| Staff worry that mistakes they make are kept in their personnel file **^b^** |
| **Feedback and communication about errors** |
| We are given feedback about changes put into place based on event reports |
| We are informed about errors that happen in this unit |
| In this unit, we discuss ways to prevent errors from happening again |
| **Overall perceptions of safety** |
| Patient safety is never sacrificed to get more work done |
| Our procedures and systems are good at preventing errors from happening |
| It is just by chance that more serious mistakes don’t happen around here **^b^** |
| We have patient safety problems in this unit **^b^** |
| **Teamwork across care units** |
| There is good cooperation among care units that need to work together |
| Care units work well together to provide the best care for patients |
| Care units do not coordinate well with each other **^b^** |
| It is often unpleasant to work with staff from other care units **^b^** |
| **Handoffs and transitions among care units** |
| Things “fall between the cracks” when transferring patients from one unit to another **^b^** |
| Important patient care information is often lost during shift changes **^b^** |
| Problems often occur in the exchange of information across care units **^b^** |
| Shift changes are problematic for patients in this care unit **^b^** |
| **Organizational learning—continuous improvement** |
| We are actively doing things to improve patient safety |
| Mistakes have led to positive changes here |
| After we make changes to improve patient safety, we evaluate their effectiveness |
| **Management support for patient safety** |
| Management provides a work climate that promotes patient safety |
| The actions of management show that patient safety is a top priority |
| Management seems interested in patient safety only after an adverse event happens **^b^** |

**^a^** Each item was based on a five-point Likert scale in which the two lowest response categories were combined into the category “disagree” and the two highest response categories into the category “agree.” A higher percentage of positive scores indicates a better patient safety culture.

**^b^** Negatively worded.
