# Supplementary material for: Patient safety culture in home care settings in Sweden: a cross-sectional survey among home care professionals
Source: BMC Health Serv Res. 2023 Sep 16;23:998. doi: 10.1186/s12913-023-10010-y (PMC10505324; doi:10.1186/s12913-023-10010-y)
Supplement: Supplementary file 2 — Additional file 2. Correlation matrix. [file 12913_2023_10010_MOESM2_ESM.docx]

**Additional file 2**

**Correlation matrix**

|  | 1 | 2 | 3 | 4 | 5 | 6 | 7 | 8 | 9 | 10 |
| --- | --- | --- | --- | --- | --- | --- | --- | --- | --- | --- |
| 2 | -0.12  (-0.35 to 0.13)  p = 0.35 |  |  |  |  |  |  |  |  |  |
| 3 | 0.21  (-0.03 to 0.43)  p = 0.09 | 0.20  (-0.04 to 0.43)  p = 0.11 |  |  |  |  |  |  |  |  |
| 4 | 0.25  (0.00 to 0.47)  p = 0.046 | 0.33  (0.09 to 0.53)  p <0.01 | 0.07  (-0.18 to 0.31)  p = 0.60 |  |  |  |  |  |  |  |
| 5 | 0.48  (0.27 to 0.65)  p < 0.01 | 0.11  (-0.14 to 0.35)  p = 0.39 | 0.05  (-0.20 to 0.30  p = 0.68 | 0.42  (0.19 to 0.61)  p < 0.01 |  |  |  |  |  |  |
| 6 | -0.31  (-0.51 to -0.07  p = 0.01 | 0.51  (0.30 to 0.67)  p < 0.01 | 0.24  (-0.00 to 0.46)  p = 0.05 | 0.16  (-0.09 to 0.39)  p = 0.21 | -0.10  (-0.34 to 0.15)  p = 0.43 |  |  |  |  |  |
| 7 | 0.35  (0.12 to 0.55)  p < 0.01 | 0.35  (0.11 to 0.55)  p <0.01 | 0.43  (0.21 to 0.61)  p < 0.01 | 0.38  (0.14 to 0.57)  p < 0.01 | 0.31  (0.06 to 0.52)  p = 0.02 | -0.03  (-0.27 to 0.22)  p = 0.83 |  |  |  |  |
| 8 | 0.06  (-0.20 to 0.31)  p = 0.65 | 0.36  (0.12 to 0.56)  p <0.01 | 0.32  (0.07 to 0.53)  p = 0.01 | 0.14  (-0.12 to 0.38)  p = 0.30 | 0.02  (-0.24 to 0.27)  p = 0.91 | 0.46  (0.24 to 0.64)  p < 0.01 | 0.20  (-0.06 to 0.43)  p = 0.13 |  |  |  |
| 9 | 0.32  (0.08 to 0.53)  p = 0.01 | 0.10  (-0.17 to 0.34)  p = 0.48 | 0.14  (-0.12 to 0.38)  p = 0.28 | 0.34  (0.10 to 0.55)  p < 0.01 | 0.14  (-0.12 to 0.39)  p = 0.28 | -0.11  (-0.36 to 0.14)  p = 0.39 | 0.30  (0.05 to 0.51)  p = 0.02 | 0.26  (0.00 to 0.48)  p = 0.048 |  |  |
| 10 | -0.15  (-0.38 to 0.10)  p = 0.25 | 0.28  (0.04 to 0.50)  p = 0.03 | 0.40  (0.17 to 0.58)  p < 0.01 | 0.11  (-0.14 to 0.35)  p = 0.38 | -0.18  (-0.41 to 0.07)  p = 0.15 | 0.65  (0.47 to 0.77)  p < 0.01 | 0.16  (-0.09 to 0.39)  p = 0.21 | 0.35  (0.11 to 0.56)  p = 0.01 | -0.19  (-0.42 to 0.07)  p = 0.16 |  |
| 11 | 0.01  (-0.25 to 0.26)  p = 0.10 | 0.23  (-0.03 to 0.46)  p = 0.08 | 0.19  (-0.07 to 0.43)  p = 0.14 | 0.24  (-0.02 to 0.47)  p = 0.07 | -0.03  (-0.28 to 0.23)  p = 0.84 | 0.42  (0.18 to 0.61)  p < 0.01 | 0.10  (-0.16 to 0.34)  p = 0.47 | 0.42  (0.18 to 0.61)  p < 0.01 | 0.07  (-0.19 to 0.32)  p = 0.62 | 0.58  (0.37 to 0.73)  p < 0.01 |

The Pearson correlation coefficient (95% CI)

1: Staffing resources

2: Communication openness

3: Teamwork within care units

4: Supervisor/manager expectations and actions promoting safety

5: Non-punitive response to error

6: Feedback and communication about error

7: Overall perceptions of safety

8: Teamwork across care units

9: Handoffs and transitions among care units

10: Organizational learning—continuous improvement

11: Management support for patient safety
